# Define a good prognosis of *RNF43* codon 659-mutated and concomitant genomic signatures in CRC: an analysis of the cBioPortal database

**DOI:** 10.3389/fonc.2025.1608664

**Published:** 2025-08-08

**Authors:** Feng Wang, Li Lin, Zhongkang Li, Lei Qin, Shuai Zhang, Xueqing Hu, Yunbo Zhao, Yingying Huang

**Affiliations:** ^1^ Department of Gastrointestinal Surgery, Beijing Tsinghua Changgung Hospital, School of Clinical Medicine, Tsinghua University, Beijing, China; ^2^ Department of Oncology Center, Peking University International Hospital, Beijing, China; ^3^ Geneplus-Beijing, Beijing, China; ^4^ Department of Oncology, Beijing Hospital, National Center of Gerontology, Beijing, China

**Keywords:** colorectal cancer (CRC), cbioportal database, RNF43-mutated, mutation analysis, prognostic

## Abstract

**Background:**

Heterogeneity of colorectal cancer (CRC) leads to significant differences in Overall Survival (OS). *RNF43* is a new predictive marker for prognosis and anti-*BRAF*/*EGFR* combinatory therapies of CRC recently. However, few studies focused on the relationship between *RNF43* and co-mutation characteristics and prognosis. This study aims to explore the different prognostic subtypes of *RNF43*-mutated CRC by analyzing the association of clinicopathological and genomic characteristics with survival outcomes.

**Methods:**

The clinical characteristics, mutational characteristics, and survival data of CRC patients were obtained for *RNF43*-mutated analysis from cBioPortal. All mutation data were filtered by the 1021-panel (Geneplus-Beijing, China), and the processed data were used to analyze the predictive value of *RNF43*-mutated to OS and concomitant co-mutations. Cox regression analysis was selected to explore prognostic biomarkers, and finally, *BRAF* and MSI were selected for subgroup analysis. The independent validation cohort comprised 339 cases of stage IV CRC from Beijing Hospital.

**Results:**

11 datasets with 4028 patient data were screened for this study. The most common variant was frameshift, which occurred in codon 659-mutated of exon 9, including *RNF43* p.G659Vfs*41 (N=116) and *RNF43* p.G659Sfs*87 (N=2). *RNF43* codon 659-mutated occurred frequently in right-sided CRC (59.32%, N=70, P<0.0001), and rarely in the left-sided (11.02%, N=13). The incidence of TMB-H in the *RNF43* codon 659-mutated group was 93.22% (110/118), and MSI-H was 78.81% (93/118). Univariate Cox analysis and multivariate Cox analysis showed that MSI-H was the most significantly different biomarker for better prognosis (P=0.004, HR=3, CI 1.4-6.4), and Class 1 *BRAF* V600E was the most different biomarker for worse prognosis (P<0.001, HR=0.3, CI 0.21-0.42). *RNF43* codon 659-mutated with non-class 1 *BRAF*-mutated or MSI-H suggests a better prognosis in CRC. We found that G1 (*RNF43* codon 659-mutated, non-class 1 *BRAF*-mutated, and MSl-H) had a better PFS and OS. The mutation difference analysis showed that the core genes related to the cancer signaling pathway (PI3K-Akt signaling pathway, MicroRNAs pathway, DNA damage repair, and tumor suppressor genes) were highly frequent in G1. The analysis comparing the core gene mutation difference between *RNF43*-mutated and wild-type in the validation cohort yielded consistent conclusions.

**Conclusions:**

In CRC, we found that the G1 cohort had the best prognosis, and patients with *RNF43* Non-codon 659-mutated, *BRAF* V600E and MSS had the worst prognosis. This may provide clinical value for patients’ further accurate prognosis prediction, curative effect prediction, and follow-up management of patients.

## 1 Introduction

Colorectal cancer (CRC) is the third most common cancer in the world and the second leading cause of cancer-related death (1). Despite considerable advances in treatment strategies and survival, the prognosis for patients with colorectal cancer remains poor, with 5-year overall survival (OS) for metastatic colorectal cancer of about 14%. The 5-year survival rate for all colorectal cancer patients is about 65% (2). Currently, the Tumor-Node-Metastasis system (TNM) classification at diagnosis is a major determinant of survival, but CRC is a highly heterogeneous disease with different molecular characteristics, including genetic and epigenetic changes (3, 4). Even when shared with the same pathological type or disease stage, there are also significant differences in treatment efficacy and survival, as well as substantial differences in the response of patients with different molecular characteristics to the same treatment strategy, leading to imprecise prognostic predictions (5–7). Therefore, predicting the survival of CRC needs further exploration.

The Wnt/β-catenin signaling pathway is a traditional pathway initiated by changes in Wnt ligand-dependent genes (*RNF43*/*ZNRF3*/*RSPO*) or ligand-independent genes (*APC*) and plays a key role in the initiation, advancement, and metastasis of CRC (8). *RNF43* (Ring finger protein 43) is an E3 ubiquitin-protein ligase that inhibits overactivation of the Wnt pathway, and *RNF43* mutations lead to permanent activation of the Wnt pathway in cancer cells (9, 10). Previous studies have reported the clinical significance of *RNF43* mutations in colorectal cancer. However, the effect of *RNF43*-mutated in colorectal cancer remains controversial. It has been suggested that *RNF43*-mutated can be used as a predictive biomarker of anti-*BRAF*/*EGFR* combination therapy response in microsatellite-stabilized (MSS) *BRAF* V600E metastatic colorectal cancer patients, and *RNF43*-mutated is a better predictive biomarker of progression-free survival (PFS) and OS in *BRAF*-mutated CRC patients (9, 11). Other studies have associated *RNF43*-mutated with poor prognosis and a higher recurrence rate (12, 13). Therefore, the prognostic value of *RNF43*-mutated remains to be determined.

*BRAF* is the core gene of the Mitogen-Activated Protein Kinase (MAPK) signaling pathway, which regulates cell proliferation and apoptosis (14). The incidence of *BRAF*-mutated CRC is about 10-20% (15, 16). Class 1 *BRAF* V600E-mutated is caused by c.1799T>A, suggesting the worst tumor biological behavior and poor prognosis, accounting for 90% of all *BRAF*-mutated in CRC according to a deeper classification system of *BRAF*-mutated derived from pre-clinical models functional studies (17, 18). *BRAF* V600 CRC has previously been extensively studied, and tumors with *RNF43*-mutated are associated with a high frequency of *BRAF* V600E-mutated, and these co-mutations are associated with poor survival (19, 20).

High microsatellite instability (MSI-H) of colon cancer can indicate better clinical outcomes of immune checkpoint inhibitors (ICIs) in the early-stage to the advanced population, and its predictive value in advanced CRC has been approved by the National Comprehensive Cancer Network (NCCN) clinical guidelines (21, 22). Previous studies have shown that *RNF43*-mutated is most associated with MSI-H, and it has been reported that *RNF43* p. G659fs* is enriched in MSI-H cancer (23, 24). These results suggest that *RNF43* is a predictive prognostic marker for colorectal cancer, and limited data are available to predict the significance of individual changes. However, a few studies on the relationship between *RNF43* and co-mutation characteristics and prognosis, and the clinical significance of *RNF43*-mutated and other biomarkers such as *BRAF* and MSI status in colorectal cancer are still worth exploring.

In this study, we explored prognostic biomarkers with predictive value based on clinicopathological and molecular characteristics of colorectal cancer patients in the cBioPortal database. We analyzed the association of *RNF43*-mutated, co-occurring mutations, genomic characteristics (including MSI, TMB), and OS, and found that *RNF43* codon 659-mutated has prognostic value and is a special subtype. We then determined the predictive prognostic value of three indicators based on *RNF43*, *BRAF*, and MSI status and performed differential mutation analysis and pathway enrichment analysis. The results reveal that the *RNF43* subtype, combined with other molecular characteristics, can be used as biomarkers to predict the clinical outcome of CRC.

## 2 Materials and methods

### 2.1 Data source and patient selection

**Figure 1:**
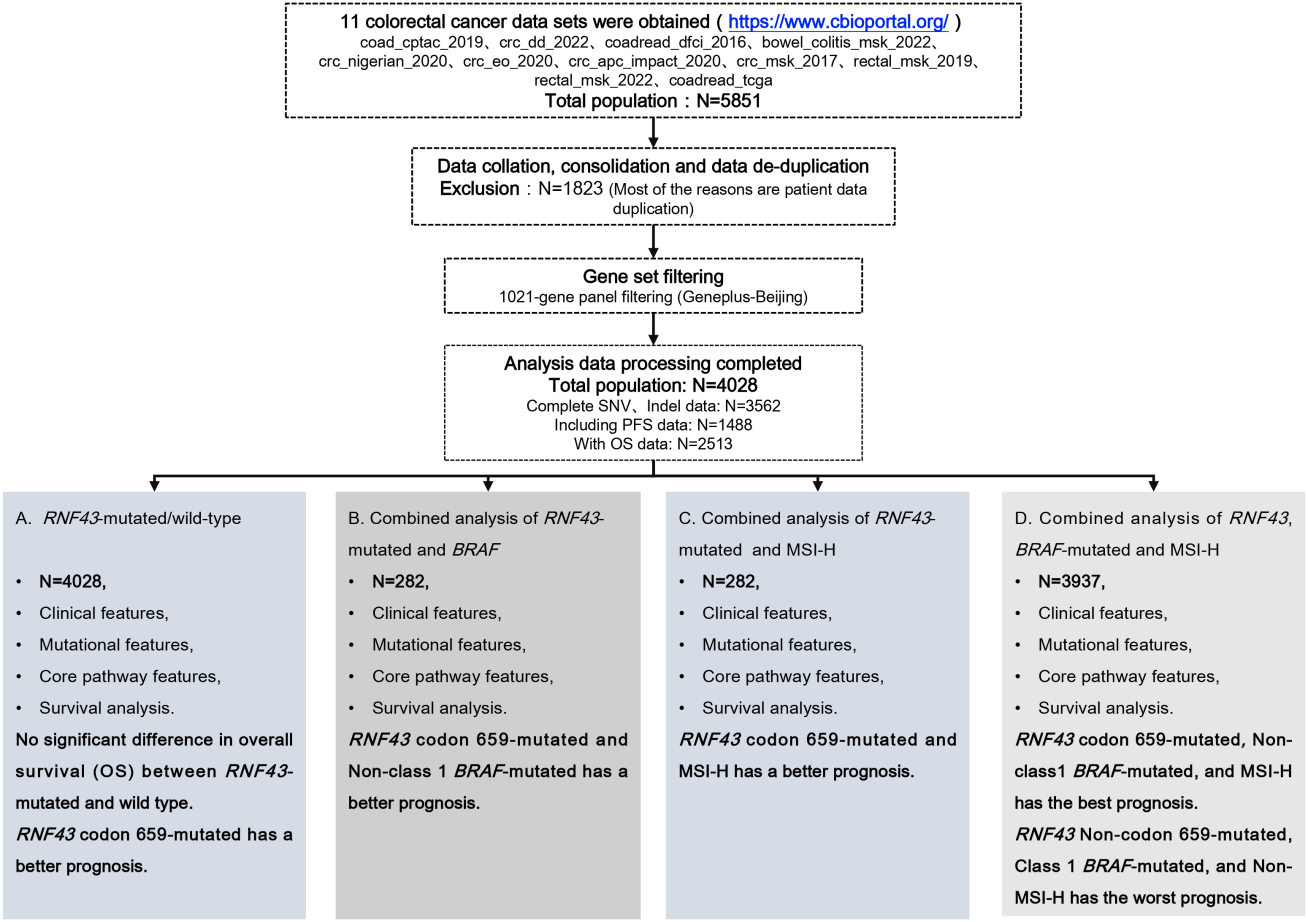
Overview of the study design.

The clinical characteristics, mutational characteristics, and survival data of CRC patients were recruited for *RNF43*-mutated analysis from the Cancer Genome Atlas (TCGA) database using the cBio Cancer Genomics Portal (cBioPortal), available at http://www.cbioportal.org (**Supplementary Tables 1**, **2**) (25). We integrated all the data sets that have been published so far. We performed data consolidation and de-duplication, excluding a total of 1823 patient data, and finally obtained 4028 patients from 11 data sets (coad_cptac_2019,crc_dd_2022,coadread_dfci_2016,bowel_colitis_msk_2022,crc_nigerian_2020,crc_eo_2020,crc_apc_impact_2020,crc_msk_2017,rectal_msk_2019,rectal_msk_2022,coadread_tcga) for this study (**Figure 1**). We then filtered single-nucleotide variants (SNVs) through the 1021 panel (Geneplus-Beijing, China), a custom-designed biotinylated oligonucleotide probe (Roche NimbleGen, Madison, WI, USA) covering ~1.4 Mbp coding region of genomic sequence of 1,021 cancer-related genes to explore the relationship with tumor genomic characteristics and prognosis (**Supplementary Table 3**). The TMB was defined as the total number of mutations per megabase (1 Mb) of non-synonymous single-nucleotide variants (SNV), insertion/deletion (Indel), and splice ±2 (26). The upper quartile of tumor mutational burden (TMB) was deemed as high TMB (TMB-H), with a threshold of 8.87 mutations/Mb in this study (27, 28). MSI-H directly used the downloaded label with cBioPortal, and the total number of MSI-H and MSS patients was 296 and 2858, respectively. Before analyzing this study, we calculated the mutation frequencies in 11 cohorts to better understand the reproducibility and limitations of the research, as shown in **Supplementary Table 4**. We evaluated the overall research results based on the completeness of data for 100 genes, 200 genes, and 300 genes, with missing rates of 6.09%, 9.09%, and 14.91%, respectively. We believe that these data not only support the overall reliability of the data but also indicate that the data has certain stability and reproducibility. A total of 339 patients with advanced CRC who underwent 1021 panel NGS sequencing served as the validation cohort for this study (**Supplementary Table 5**) (29). The study was conducted in accordance with the Declaration of Helsinki (as revised in 2013). The study was approved by the Ethics Committee of Beijing Hospital (2023BJYYEC-428-02).

### 2.2 Statistical analysis

All the data were analyzed using the R statistics package (R version 4.2.1, Austria) or GraphPad Prism version 8 (GraphPad Software, CA, USA). Differences between designed groups were analyzed based on the Fisher test or the t-test. Univariate Cox regression and Multivariate Cox regression analysis methods were used to analyze the correlation between mutation characteristics, genomic characteristics, and clinical outcomes. David 6.8 (https://david.ncifcrf.gov/) was used to carry out the Gene Ontology (GO) and Kyoto Encyclopedia of Genes and Genomes (KEGG) pathway enrichment analysis. The log-rank test Kaplan-Meier (KM) survival curve was used to calculate prognostic differences between groups based on *RNF43*-mutated. Survival curves were calculated by the Kaplan-Meier method, and differences between groups based on *RNF43*-mutated status were tested by the log-rank test. P values < 0.05 were denoted as statistically significant.

## 3 Results

### 3.1 Patient characteristics

The clinicopathological and molecular characteristics of the enrolled patient population are shown in **Table 1**. The mean age of this cohort was 57.94 years, and most of the population (41.91%, N=1688) was between 50 and 70 years old. At the primary tumor site, the *RNF43*-mutated group had significantly more right-sided patients than left-sided patients (left-sided: 24.47%, N=69; right-sided: 52.13%, N=147), and the *RNF43* wild-type cohort data were contrary (left-sided: 54.30%, N=2034; right-sided: 22.02%, N=825). A total of 65% of the patients in the whole cohort were stage III-IV patients, and the tumor grade was mainly moderately differentiated (34.01%, N=1370). The genomic markers TMB-H (198/282, 70.21%, P<0.001) and MSI-H (138/282, 48.94%, P<0.001) were significantly higher in the *RNF43*-mutated group compared to those in the *RNF43* wild-type group. Class 1 *BRAF*-mutated and *RNF43*-mutated co-occurred frequently. In the *RNF43*-mutated group, the proportion of class 1 *BRAF*-mutated was 34.05%, and that in the *RNF43* wild-type group was only 7.13%.

**Table 1:** Clinicopathological and molecular characteristics of this study.

Clinicopathologic characteristics	Number of patients, N (%) (N=4028)	*RNF43* mut, N (%) (N=282)	*RNF43* wild-type, N (%) (N=3746)
Age(median 57.94, range 13–95)			
Young (years <50)	1321 (32.80%)	83 (29.43%)	1238 (33.05%)
Intermediate (<70 years ≥50)	1688 (41.91%)	103 (36.52%)	1585 (42.31%)
Elder (years≥70)	991 (24.60%)	95 (33.69%)	896 (23.92%)
NA	28 (0.70%)	1 (0.35%)	27 (0.72%)
Gender			
Female	1895 (47.05%)	149 (52.84%)	1746 (46.61%)
Male	2071 (51.42%)	118 (41.84%)	1953 (52.14%)
NA	62 (1.54%)	15 (5.32%)	47 (1.25%)
Primary tumor location			
Right	972 (24.13%)	147 (52.13%)	825 (22.02%)
Left	2103 (52.21%)	69 (24.47%)	2034 (54.30%)
NA	953 (23.66%)	66 (23.40%)	887 (23.68%)
TNM stage			
I	435 (10.80%)	38 (13.48%)	397 (10.60%)
II	769 (19.09%)	91 (32.27%)	678 (18.10%)
III	1252 (31.08%)	77 (27.30%)	1175 (31.37%)
IV	1400 (34.76%)	56 (19.86%)	1344 (35.88%)
NA	172 (4.27%)	20 (7.09%)	152 (4.06%)
TUMOR_GRADE			
Well differentiated	525 (13.03%)	45 (15.96%)	480 (12.81%)
Moderately differentiated	1370 (34.01%)	63 (22.34%)	1307 (34.89%)
Moderate poorly differentiated	114 (2.83%)	10 (3.55%)	104 (2.78%)
Poorly differentiated	355 (8.81%)	58 (20.57%)	297 (7.93%)
NA	1664 (41.31%)	106 (37.59%)	1558 (41.59%)
TMB			
TMB-H	921 (22.86%)	198 (70.21%)	723 (19.30%)
TMB-L	2765 (68.64%)	67 (23.76%)	2698 (72.02%)
NA	342 (8.49%)	17 (6.03%)	325 (8.68%)
MSI			
MSI-H	296 (7.35%)	138 (48.94%)	158 (4.22%)
MSS	2858 (70.95%)	92 (32.62%)	2766 (73.84%)
NA	874 (21.70%)	52 (18.44%)	822 (21.94%)
*BRAF* status			
*BRAF* mut	363 (9.01%)	96 (34.04%)	267 (7.13%)
*BRAF* wild-type	3665 (90.99%)	186 (65.96%)	3479 (92.87%)
*BRAF* mutation types			
Class 1	256 (6.36%)	86 (30.50%)	170 (4.54%)
Class 2	9 (0.22%)	0 (0.00%)	9 (0.24%)
Class 3	40 (0.99%)	0 (0.00%)	40 (1.07%)
NA	58 (1.44%)	10 (3.55%)	48 (1.28%)

### 3.2 The Landscape of the *RNF43*-mutated CRC

**Figure 2:**
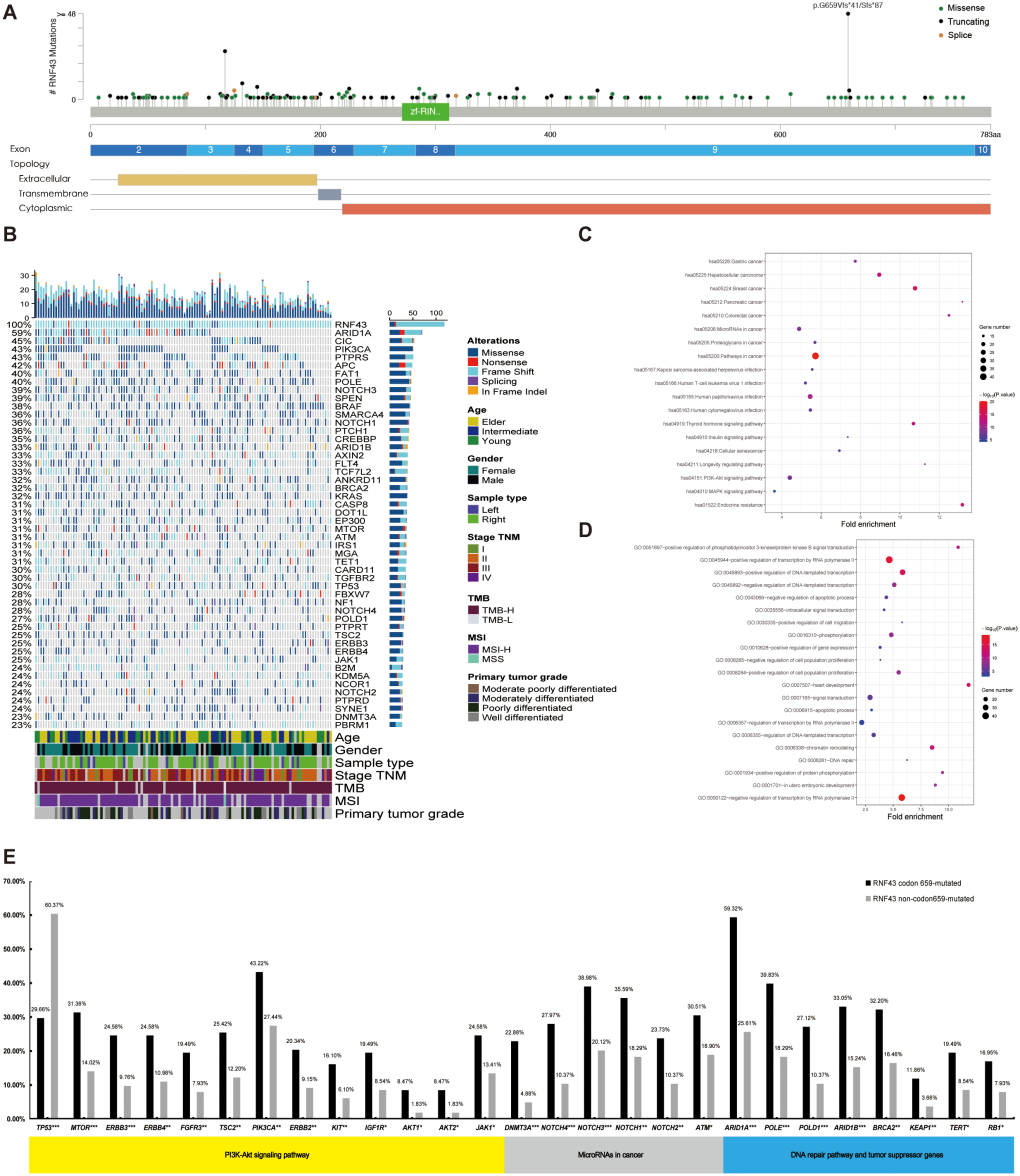
A panoramic analysis of the genomic and pathway characteristics of *RNF43*-mutated in CRC. **(A)** Lollipop plots (maps mutations on a linear protein and its domains) in this study. Truncating includes frameshift mutations and nonsense mutations. **(B)** Top 50 mutation spectrum in 283 *RNF43*-mutated patients. Each column represents a patient, and each row represents a gene. The table on the left represents the mutation rate of each gene. The top plot represents the overall number of mutations a patient carried. Different colors denote different types of mutations. KEGG **(C)** and GO **(D)** functional enrichment analyses of *RNF43*-mutated. GO, Gene Ontology; KEGG, Kyoto Encyclopedia of Genes and Genomes. **(E)** The differences in core gene mutation of major signaling pathways (PI3K-Akt signaling pathway, MicroRNAs pathway, DNA damage repair, and tumor suppressor genes) between *RNF43* codon 659-mutated and *RNF43* Non-codon 659-mutated. CRC, Colorectal cancer; *p<0.05; **p<0.01; ***p<0.001.

In this study, 375 *RNF43* variants were detected in 282 *RNF43*-mutated patients. The most common variant was frameshift, which occurred in codon 659-mutated of exon 9, including *RNF43* p. G659Vfs*41 (N=116) and *RNF43* p. G659Sfs*87 (N=2), as shown in **Figure 2A**, **Supplementary Table 6**. The distribution range of *RNF43*-mutated varies (1.89%-28.13%) in 11 cohorts. The frequency of *RNF43*-mutated in the vast majority of cohorts was between 7.34% and 17.27%. Additionally, *RNF43* codon 659-mutated has a relatively high proportion in all queues, including coadread_dfci_2016, cro_eo_2020, rectal_msk_2022, and coad_cptac_2019 (**Supplementary Figure 1**). In *RNF43*-mutated cohorts, the most commonly mutated genes were *ARIDIA* (59%), *CIC* (45%), *PIK3CA* (43%), *PTPRS* (43%), *APC* (42%), *FAT1* (40%), *POLE* (40%), *NOTCH3* (39%), *SPEN* (39%), *BRAF* (38%) (**Figure 2B**). In contrast, the top10 mutated genes in *RNF43* wild-type tumors were *APC* (74%), *TP53* (71%), *KRAS* (41%), *PIK3CA* (17%), *FBXW7* (14%), *SMAD4* (13%), *TCF7L2* (10%), *SOX9* (10%), *ARID1A* (8%), *BRAF* (8%) (**Supplementary Figure 2A**). Survival analysis showed that *RNF43*-mutated had worse progression-free survival (PFS, P=0.0048) and overall survival (OS, P=0.18) (**Supplementary Figures 2B, C**). Considering the limitations of single-mutation data, we conducted a joint analysis using 106 mRNA data from the coad_cptac_2019 dataset. We found that the expression level of *RNF43* in the *RNF43*-mutated was significantly lower than that in the *RNF43* wild-type (P < 0.001, **Supplementary Figure 2D**). Meanwhile, we found that there were also cases of low *RNF43* expression levels within the *RNF43* wild-type. Given the relatively small size of the study cohort, we integrated two groups: the *RNF43*-mutated with expression values < 0, and the *RNF43* wild-type with expression values > 0. We found that the *RNF43*-mutated/expression < 0 shared a similar mutation spectrum with the *RNF43*-mutated, and *RNF43* wild-type/expression > 0 had a similar mutation spectrum to the *RNF43* wild-type (**Supplementary Figure 2E**). This finding implies that DNA combined with RNA-based approaches for precise prognostic stratification represents a more optimal choice in the future.

In the validation cohort, we found consistent results in the *RNF43*-mutated: *KRAS* (32% vs 43%), *APC* (42% vs 35%), *ARID1A* (59% vs 35%), and *NF1* (35% vs 28%). However, there were differences in *TP53*. The abundance in the validation cohort is as high as 70%. The top mutations of the *RNF43* wild-type showed high consistency in both the analysis cohort and the validation cohort (**Supplementary Figures 3A, B**). Differential gene analysis showed that the *RNF43*-mutated group had significantly higher mutation frequency (**Supplementary Table 7**). The mutation differences between *RNF43*-mutated and *RNF43* wild-type in validation cohort was also analyzed. We founf that *NF1, ARID1A, BRAF, B2M, WRN* were significantly enriched in *RNF43*-mutated group, while *APC* was significantly enriched in *RNF43* wild-type, and the *RNF43*-mutated group had significantly higher mutation frequency (**Supplementary Table 8**, **Supplementary Figure 3C**). KEGG pathway enrichment analysis showed that hsa05206: MicroRNAs in cancer, and hsa04151: PI3K-Akt signaling pathway were significantly enriched in the *RNF43*-mutated group (**Figure 2C**). GO enrichment analysis showed that the *RNF43*-mutated group had higher enrichment of proliferative signaling pathway (GO: 0016310-Phosphorylation, GO: 0008284~positive regulation of cell population proliferation, GO: 0043066-negative regulation of apoptotic process, etc) (**Figure 2D**). The results of KEGG and GO enrichment analyses of the verification cohort were consistent (**Supplementary Figures 3D, E**).

### 3.3 *RNF43* codon 659-mutated is a specific subtype of CRC

As the incidence of codon 659 mutation accounted for nearly half of the total *RNF43*-mutated and had unique clinical significance in predicting the efficacy of anti-*BRAF*/*EGFR* combinatory therapies (30), the p.G659Vfs*41 and p.G659Sfs*87 was defined as the *RNF43* codon 659-mutated group and the other mutation types were defined as the *RNF43* Non-codon 659-mutated group in this study. The clinicopathological and molecular characteristics of the two groups were different from the total population (**Table 2**). The proportion of *RNF43* codon 659-mutated patients aged over 70 years was higher (38.98% vs. 29.88%, P=0.2249). The *RNF43* Non-codon 659-mutated occurs most frequently in 50-70 years. There was no difference in gender between the two groups. *RNF43* codon 659-mutated occurred frequently in right-sided CRC (59.32%, N=70, P<0.0001), and rarely in the left-sided (11.02%, N=13), while the left and right-sided were more balanced in the *RNF43* Non-codon 659-mutated (34.15%, N=56; 46.95%, N=77). In terms of TNM stage, *RNF43* codon 659-mutated mainly appeared in TNM II-III (66.1%, N=78), which was inconsistent with the total group staging concentrated in III-IV (65.85%, N=2652).

**Table 2:** Clinicopathological and molecular characteristics of *RNF43* codon 659-mutated and *RNF43* Non-codon 659-mutated.

Clinicopathologic characteristics	Number of patients, N (%) (N=282)	*RNF43* codon 659-mutated, N (%) (N=118)	*RNF43* Non-codon 659-mutated, N (%) (N=164)	P value
Age (median 57.94, range 13–95)				0.2249
Young (years <50)	83 (29.43%)	30 (25.42%)	53 (32.32%)	
Intermediate (<70 years ≥50)	103 (36.52%)	41 (34.75%)	62 (37.80%)	
Elder (years≥70)	95 (33.69%)	46 (38.98%)	49 (29.88%)	
NA	1 (0.35%)	1 (0.85%)	0 (0.00%)	
Gender				0.1343
Female	149 (52.84%)	60 (50.85%)	89 (54.27%)	
Male	118 (41.84%)	48 (40.68%)	70 (42.68%)	
NA	15 (5.32%)	10 (8.47%)	5 (3.05%)	
Primary tumor location				<0.0001
Right	147 (52.13%)	70 (59.32%)	77 (46.95%)	
Left	69 (24.47%)	13 (11.02%)	56 (34.15%)	
NA	66 (23.40%)	35 (29.66%)	31 (18.90%)	
TNM stage				0.0124
I	38 (13.48%)	12 (10.17%)	26 (15.85%)	
II	91 (32.27%)	43 (36.44%)	48 (29.27%)	
III	77 (27.30%)	35 (29.66%)	42 (25.61%)	
IV	56 (19.86%)	15 (12.71%)	41 (25.00%)	
NA	20 (7.09%)	13 (11.02%)	7 (4.27%)	
TUMOR_GRADE				
Well differentiated	45 (15.96%)	14 (11.86%)	31 (18.90%)	**0.0196**
Moderately differentiated	63 (22.34%)	19 (16.10%)	44 (26.83%)	
Moderate poorly differentiated	10 (3.55%)	3 (2.54%)	7 (4.27%)	
Poorly differentiated	58 (20.57%)	26 (22.03%)	32 (19.51%)	
NA	106 (37.59%)	56 (47.46%)	50 (30.49%)	
TMB				<0.0001
TMB-H	198 (70.21%)	110 (93.22%)	88 (53.66%)	
TMB-L	67 (23.76%)	2 (1.69%)	65 (39.63%)	
NA	17 (6.03%)	6 (5.08%)	11 (6.71%)	
**MSI**				**<0.0001**
MSI-H	138 (48.94%)	93 (78.81%)	45 (27.44%)	
MSS	92 (32.62%)	2 (1.69%)	90 (54.88%)	
NA	52 (18.44%)	23 (19.49%)	29 (17.68%)	
*BRAF* status				0.2519
*BRAF* mut	96 (34.04%)	45 (38.14%)	51 (31.10%)	
*BRAF* wild-type	186 (65.96%)	73 (61.86%)	113 (68.90%)	
*BRAF* mutation types				0.3272
Class 1	86 (30.50%)	42 (35.59%)	44 (26.83%)	
Class 2	0 (0.00%)	0 (0.00%)	0 (0.00%)	
Class 3	0 (0.00%)	0 (0.00%)	0 (0.00%)	
NA	10 (3.55%)	3 (2.54%)	7 (4.27%)	

Subsequently, we analyzed the mutation differences between *RNF43* codon 659-mutated and *RNF43* Non-codon 659-mutated and pathway enrichment results. *CIC*, *ARID1A*, *PTCH1*, *SMARCA4*, *FLT4* were significantly enriched in *RNF43* codon 659-mutated group, while *TP53* was significantly enriched in *RNF43* Non-codon 659-mutated (**Supplementary Table 8**). The differences in core gene mutation of major signaling pathways (PI3K-Akt signaling pathway, MicroRNAs pathway, DNA damage repair, and tumor suppressor genes) are shown in **Figure 2E**. Except for *TP53* has the highest frequency in the *RNF43* wild-type, other frequencies are significantly higher in *RNF43*-mutated. Considering that the population of RNF43 codon 659-mutated in the validation cohort is relatively small (only three cases), we are temporarily unable to carry out the validation work for this part.

### 3.4 Class 1 *BRAF*-mutated and MSI-H have strong prognostic value in CRC

**Figure 3:**
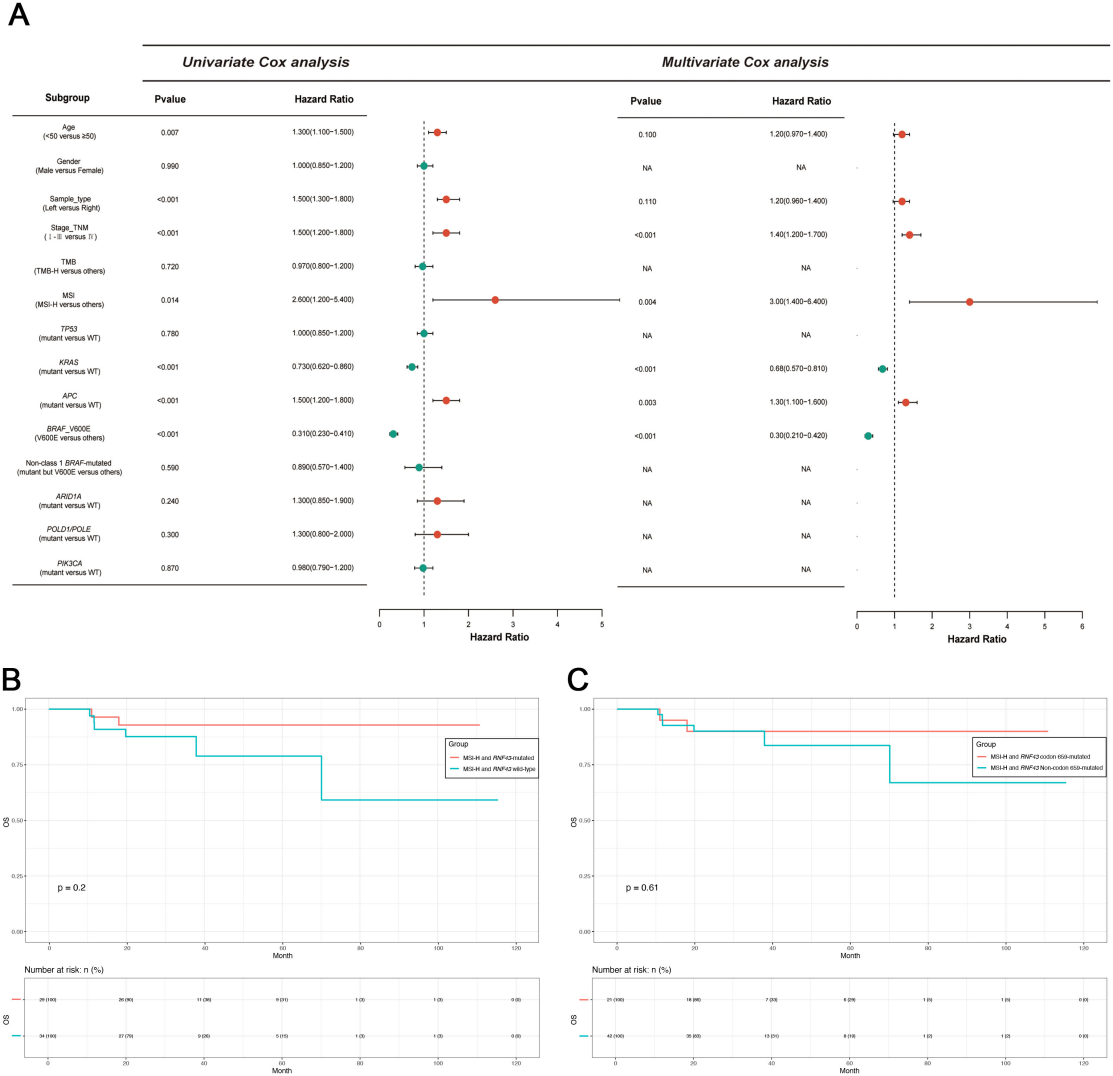
Univariate and multivariate Cox proportional hazards analysis of OS and survival analysis of the MSI-H subgroup in this study. **(A)** Univariate and multivariate Cox proportional hazards analysis of OS in this study. **(B)** Survival analysis of MSI-H with or without *RNF43.* **(C)** Survival analysis of MSI-H with or without *RNF43* codon 659-mutated. OS, overall survival; TMB-H, patients with high TMB; MSI-H, patients with high MSI; WT, wild type.

Based on the differences in clinical features and mutational characteristics exhibited by *RNF43* codon 659-mutated and *RNF43* Non-codon 659-mutated. Our next step aims to screen biomarkers that predict prognosis. We conducted univariate Cox analysis and multivariate Cox analysis based on OS as clinical outcomes. Study results are shown in **Figure 3A**. We found age, sample_type (left-sided or right-sided), stage_TNM, MSI, *KRAS*, *APC*, and *BRAF*_V600E (Class1 *BRAF*-mutated) were biomarkers with significant prognostic differences. Subsequently, factors with P<0.05 were included in multivariate analysis, and it was found that MSI-H was the most significantly different biomarker for better prognosis (P=0.004, HR=3, CI 1.4-6.4), and Class 1 *BRAF* V600E was the most different biomarker for worse prognosis (P<0.001, HR=0.3, CI 0.21-0.42). We also found that *KRAS*-mutated was the second-highest predictor of poor prognosis (P<0.001, HR=0.68, CI 0.57-0.81).

MSI-H is a molecular marker that is included in the guidelines and serves as a biomarker indicating a favorable prognosis for CRC. Therefore, further exploration on the value of *RNF43*-mutated and *RNF43* wild-type based on the MSI-H is necessary. Thus, we conducted two groups: MSI-H and *RNF43*-mutated (N=138), and MSI-H and *RNF43* wild-type (N=158). We first conducted a statistical analysis of clinical information (**Table 3**). No significant differences in age, gender, stage, and tumor grade. However, the proportion of left-sided tumors in the MSI-H and *RNF43*-mutated group was significantly lower than that in the MSI-H and *RNF43* wild-type group (15.22% vs. 28.48%, P=0.0235), and the TMB-H proportion in the MSI-H and *RNF43*-mutated group was higher (95.65% vs. 87.97%, P=0.0196). Next, we found the overall mutation frequency of MSI-H and *RNF43*-mutated was higher than that of MSI-H and *RNF43* wild-type, with a difference in the distribution of mutations (**Supplementary Table 9**). In the MSI-H and *RNF43*-mutated group, the top 5 mutations were *ARID1A, PTPRS, FAT1, PIK3CA*, and *SPEN* (**Supplementary Figure 4A**). The top 5 mutations in the MSI-H and *RNF43* wild-type group were *APC, ARID1A, PIK3CA, PTPRS*, and *BRAF* (**Supplementary Figure 4B**). Furthermore, we analyzed mutations specifically in the MSI-H and *RNF43* codon 659-mutated group compared to the MSI-H and *RNF43* Non-codon 659-mutated group. The results revealed that the high-frequency mutations in the MSI-H and *RNF43* codon 659-mutated group included *ARID1A, CIC, PTPRS, FAT1*, and *PIK3CA* (**Supplementary Figure 4C**), with a higher mutation frequency than observed in the MSI-H and *RNF43* Non-codon 659-mutated group (**Supplementary Figure 4D**). Finally, we conducted a prognostic analysis; the OS of the MSI-H and *RNF43*-mutated group was better than that of the MSI-H and *RNF43* wild-type group, but there was no significant difference (P = 0.2, **Figure 3B**). The OS of the MSI-H and *RNF43* codon 659-mutated group was better than that of the MSI-H and *RNF43* Non-codon 659-mutated group, and there was also no significant difference (P = 0.61, **Figure 3C**). This lack of significant difference may be attributed to the high proportion of poorly differentiated individuals within the MSI-H and *RNF43*-mutated cohort.

**Table 3:** Clinicopathological and molecular characteristics of MSI-H and *RNF43*-mutated or *RNF43* wild-type.

Clinicopathologic characteristics	Number of patients, N (%) (N=296)	MSI-H and RNF43-mutated, N (%) (N=138)	MSI-H and RNF43 wild-type, N (%) (N=158)	P value
Age (median 57.94, range 13–95)				0.3582
Young (years <50)	77 (26.01%)	31 (22.46%)	46 (29.11%)	
Intermediate (<70 years ≥50)	112 (37.84%)	57 (41.30%)	55 (34.81%)	
Elder (years≥70)	106 (35.81%)	49 (35.51%)	57 (36.08%)	
NA	1 (0.34%)	1 (0.72%)	0 (0.00%)	
Gender				0.8821
Female	150 (50.68%)	72 (52.17%)	78 (49.37%)	
Male	144 (48.65%)	65 (47.10%)	79 (50.00%)	
NA	2 (0.68%)	1 (0.72%)	1 (0.63%)	
Primary tumor location				0.0235
Right	180 (60.81%)	92 (66.67%)	88 (55.70%)	
Left	66 (22.30%)	21 (15.22%)	45 (28.48%)	
NA	50 (16.89%)	25 (18.12%)	25 (15.82%)	
TNM stage				0.6909
I	38 (12.84%)	17 (12.32%)	21 (13.29%)	
II	112 (37.84%)	57 (41.30%)	55 (34.81%)	
III	93 (31.42%)	38 (27.54%)	55 (34.81%)	
IV	47 (15.88%)	23 (16.67%)	24 (15.19%)	
NA	6 (2.03%)	3 (2.17%)	3 (1.90%)	
TUMOR_GRADE				0.0835
Well differentiated	70 (23.65%)	29 (21.01%)	41 (25.95%)	
Moderately differentiated	74 (25.00%)	30 (21.74%)	44 (27.85%)	
Moderate poorly differentiated	13 (4.39%)	4 (2.90%)	9 (5.70%)	
Poorly differentiated	59 (19.93%)	36 (26.09%)	23 (14.56%)	
NA	80 (27.03%)	39 (28.26%)	41 (25.95%)	
TMB				0.0196
TMB-H	271 (91.55%)	132 (95.65%)	139 (87.97%)	
TMB-L	7 (2.36%)	0 (0.00%)	7 (4.43%)	
NA	18 (6.08%)	6 (4.35%)	12 (7.59%)	
BRAF status				0.6302
BRAF mut	110 (37.16%)	49 (35.51%)	61 (38.61%)	
BRAF wild-type	186 (62.84%)	89 (64.49%)	97 (61.39%)	

### 3.5 *RNF43* codon-659-mutated, class 1 *BRAF*-mutated, MSI-H has strong co-mutational characteristics

**Figure 4:**
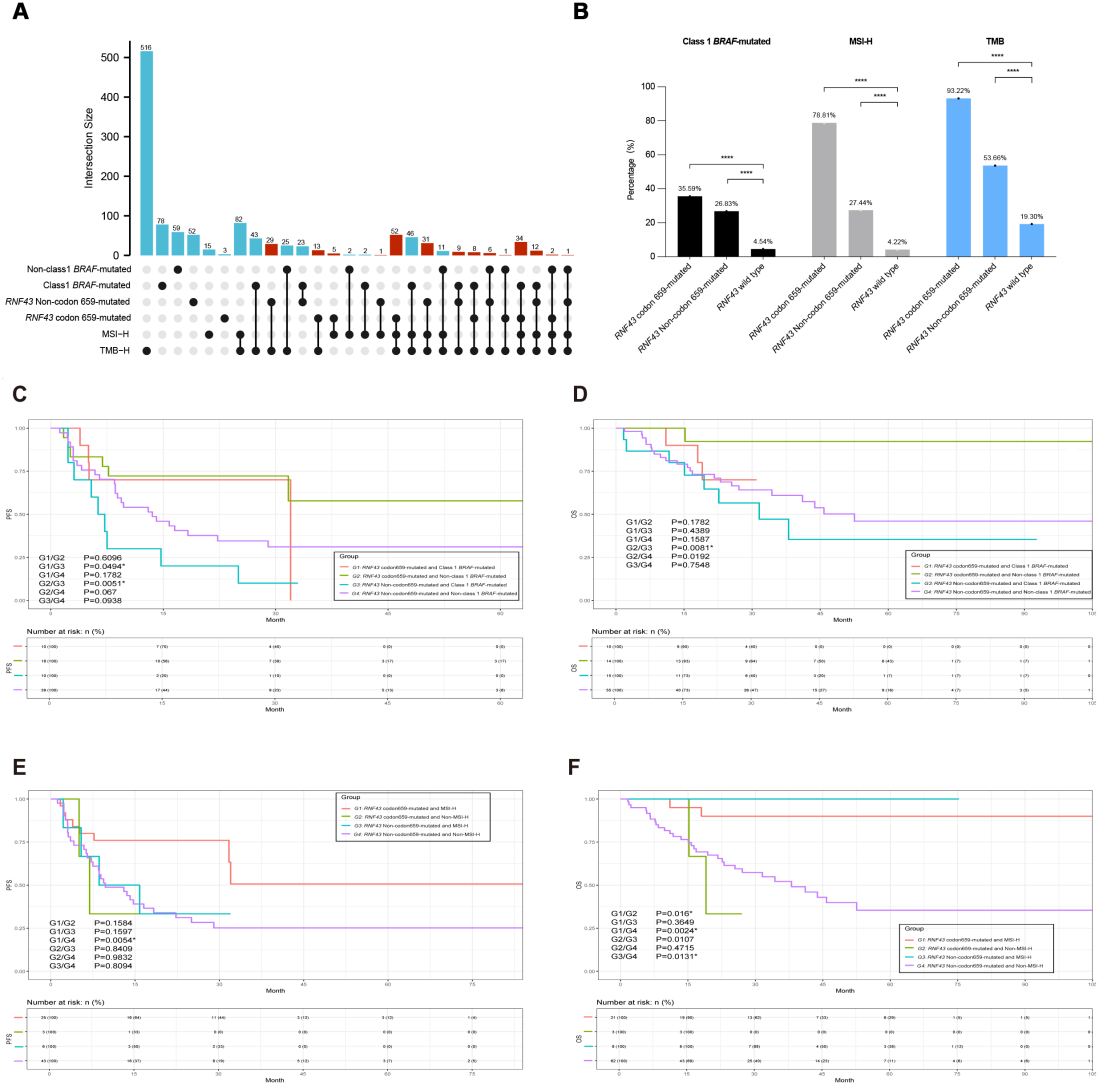
The integrated association’s analysis and survival analysis of *RNF43* codon 659-mutated, MSI status, or *BRAF*-mutated in CRC. **(A)** UPSET plot showing the shared and unique marker numbers between *RNF43*, MSI, and *BRAF* in this study. **(B)** Correlation analysis bar chart of *RNF43*, MSI, and *BRAF* indicators. *p < 0.05; **p < 0.01; ***p < 0.001; ****p < 0.0001. **(C)** KM analysis of PFS between *RNF43*-mutated and *BRAF*-mutated in this study. **(D)** KM analysis of OS between *RNF43*-mutated and *BRAF*-mutated in this study. **(E)** KM analysis of PFS between *RNF43*-mutated and MSI status in this study. **(F)** KM analysis of OS between *RNF43*-mutated and MSI status in this study. CRC, Colorectal cancer; PFS, progression-free survival; OS, overall survival; KM, Kaplan-Meier.

Further cluster analysis was performed for the prognostic markers *BRAF* and MSI, identified by Cox analysis before. We found high co-occurrence of the *RNF43* mutation subtype and *BRAF* mutation, as well as strong associations with TMB and MSI. We conducted a multi-index UPSET correlation analysis. *RNF43* codon 659-mutated was found to overlap with TMB-H, MSI-H, and *BRAF* V600E. The overlap degree of *RNF43* Non-codon 659-mutated with TMB, MSI, and *BRAF* V600E is lower than that of *RNF43* codon 659-mutated (**Figure 4A**). Class1 *BRAF*-mutated and *RNF43* codon 659-mutated, *RNF43* Non-codon 659-mutated, and *RNF43* wild-type were 35.59%, 28.83% and 4.54%, respectively (P<0.0001). It is also worth noting that the incidence of TMB-H in the *RNF43* codon 659-mutated group was 93.22% (110/118), and MSI-H was 78.81% (93/118). This suggests that the co-occurrence of *RNF43* with TMB-H or MSI-H is mainly caused by *RNF43* codon 659-mutated (**Figure 4B**, **Table 2**). Combined with the literature reporting that MSI-H is one of the factors with better prognosis in CRC (21, 22), we believe that multi-indicator association analysis may suggest a better prognosis CRC subgroup.

### 3.6 *RNF43* codon 659-mutated with non-class 1 *BRAF*-mutated or MSI-H suggests a better prognosis in CRC

We then performed a joint analysis of the two indicators, starting with *RNF43* combined with *BRAF*. A total of 282 patients with *RNF43*-mutated were enrolled and divided into four groups: G1 (N=42): *RNF43* codon 659-mutated and Class 1 *BRAF*-mutated; G2 (N=76): *RNF43* codon 659-mutated and Non-class 1 *BRAF*-mutated; G3 (N=44): *RNF43* Non-codon 659-mutated and Class 1 *BRAF*-mutated; G4 (N=120): *RNF43* Non-codon 659-mutated and Non-class 1 *BRAF*-mutated. The clinicopathological and molecular characteristics of G1-G4 groups are shown in **Supplementary Table 10**. Survival analysis of PFS results showed that the G1 group (P=0.0494) and G2 group (P=0.0051) had significantly better prognosis compared with G3 (**Figure 4C**), and OS analysis results showed that only the G2 group and G3 group had significant differences (P=0.0081, **Figure 4D**). Patients with *RNF43* codon 659-mutated and Non-class 1 *BRAF*-mutated were found to have a better prognosis. Next, we analyzed the mutation difference between the G2 group and G3 group, and the TOP mutations of the two groups were shown in **Supplementary Figure 5A** (G2 group) and **Supplementary Figure 5B** (G3 group). The volcano map of mutation difference analysis showed that *ARID1A*, *CIC*, and other genes were significantly enriched in the G2 group (**Supplementary Figure 5C**), and the pathway enrichment results suggested that the microRNAs pathway, DNA damage repair, and tumor suppressive gene mutations were significantly enriched in G2 group: *RNF43* codon 659-mutated and Non-class 1 *BRAF*-mutated. This is consistent with the results of the *RNF43-*mutated vs *RNF43* wild-type analysis, suggesting that the combined detection of *RNF43* and *BRAF* can help predict a better prognosis (**Supplementary Figures 5D-F**).

In order to match the clinical guidelines recommended, we only compared *RNF43* combined with MSI and did not perform *RNF43* combined with TMB. Therefore, four groups are assigned. G1 (N=93): *RNF43* codon 659-mutated and MSI-H; G2 (N=25): *RNF43* codon 659-mutated and Non-MSI-H; G3 (N=45): *RNF43* Non-codon 659-mutated and MSI-H; G4 (N=119): *RNF43* Non-codon 659-mutated and Non-MSI-H. This part also included 282 *RNF43*-mutated patients. The clinicopathological and molecular characteristics of the four groups are shown in **Supplementary Table 11**. As expected, G4 had worse PFS and OS (G1/G4-PFS: P=0.0054; G1/G4-OS: P=0.0024). The top mutations of group G1 and group G4 were shown in **Supplementary Figure 6A** (G1) and **Supplementary Figure 6B** (G4). Volcanic map analysis of mutation differences showed that *ARID1A*, *CIC*, *PTPRS*, *PTCH1*, and other genes were significantly enriched in group G1, and TP53 was significantly enriched in group G4 (**Supplementary Figure 6C**). This is consistent with the conclusion that CRC patients carrying *TP53* mutations have a worse prognosis. The enrichment results were consistent with the results of *RNF43-*mutated vs *RNF43* wild-type and *RNF43* combined *BRAF* analysis (**Supplementary Figures 6D–F** **).**

### 3.7 *RNF43* codon 659-mutated combined with non-class 1 *BRAF*-mutated and MSI-H has the best prognosis

**Figure 5:**
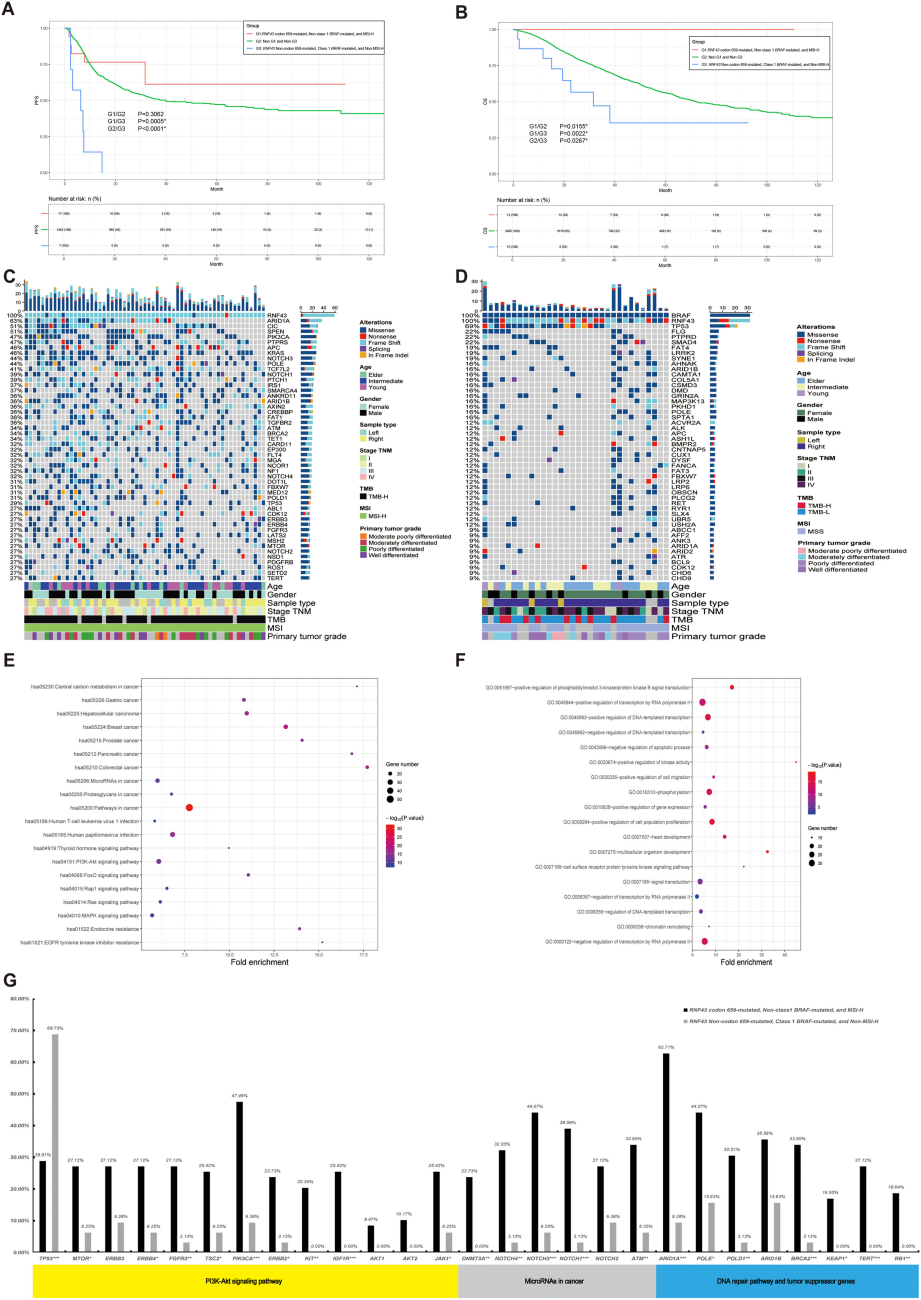
A panoramic analysis of the OS outcome, genomic and pathway characteristics of *RNF43*-mutated, MSI, and *BRAF* in CRC. **(A)** KM analysis of PFS between *RNF43*-mutated, MSI, and *BRAF*-mutated in this study. **(B)** KM analysis of OS between *RNF43*-mutated, MSI, and *BRAF*-mutated in this study. Top 50 mutation spectrum in G1 **(C)** and G3 **(D)** *RNF43*-mutated patients. Each column represents a patient, and each row represents a gene. The table on the left represents the mutation rate of each gene. The top plot represents the overall number of mutations a patient carried. Different colors denote different types of mutations. KEGG **(E)** and GO **(F)** functional enrichment analyses of G1 and G3. GO, Gene Ontology; KEGG, Kyoto Encyclopedia of Genes and Genomes. G: The differences in core gene mutation of major signaling pathways (PI3K-Akt signaling pathway, MicroRNAs pathway, DNA damage repair, and tumor suppressor genes) between G1 and G3. G1: *RNF43* codon 659-mutated, Non-class 1 *BRAF*-mutated, and MSI-H, G3: *RNF43* Non-codon 659-mutated (including *RNF43* wild-type), Class 1 *BRAF*-mutated, and Non-MSI-H; G2: Non-G1 and Non-G3. CRC, Colorectal cancer; *p<0.05; **p<0.01; ***p<0.001.

Subsequently, we integrated the three indicators of *RNF43*, *BRAF*, and MSI found above for integrated analysis, to find the population with the best prognosis. Different from the previous analysis process, we also included *RNF43* wild-type in this part, and a total of 3937 CRC patients with survival data were recorded and screened, which were divided into three groups: G1: *RNF43* codon 659-mutated, Non-class 1 *BRAF*-mutated, and MSl-H, G3: *RNF43* Non-codon 659-mutated (including *RNF43* wild-type), Class 1 *BRAF*-mutated, and Non-MSl-H; G2: Non-G1 and Non-G3. The study found that the G1 had a better PFS and the G3 had a worse PFS (G1/G3: P=0.0005; G1/G2: P=0.3062; G2/G3: P<0.0001, **Figure 5A**). The results of the OS survival analysis were more significant: the OS of G1 was 100%. The G3 has the worst OS in this cohort (G1/G2: P=0.0155; G1/G3: 0.0022; G2/G3: 0.0267, **Figure 5B**). *RNF43* codon 659-mutated, Non-class 1 *BRAF*-mutated, and MSl-H were found to have the best prognosis in the population.

Meanwhile, we performed clinicopathological analysis and mutation characteristic analysis, as shown in **Table 4**. Compared with the G2 and G3 groups, the age of G1 was higher than that of the Elder group (64.71%; G1/G2: P<0.0001; G1/G3: P<0.0001). There was no significant difference in gender among the three groups. In terms of primary tumor location, contrary to previous conclusions, G1 was significantly enriched on the right side (88.24%; G1/G2: P<0.0001; G1/G3: P<0.0001). G1 was significantly enriched in stage II colorectal cancer (52.94%; G1/G2: P<0.0001; G1/G3: P = 0.0087). In terms of Tumor_Grade, the frequency of poorly differentiated tumors was higher in G1 (38.24%, N=13), but there was no statistical difference in G1/G3 group (P=0.0767). Finally, we show the mutation landscape of G1 (**Figure 5C**) and G3 (**Figure 5D**) and carry out mutation difference analysis (**Supplementary Table 12**) and pathway enrichment (**Figures 5E–F**). The enrichment results of major pathways were consistent with the results of *RNF43*-mutated/*RNF43* wild-type and *RNF43*-mutated and *BRAF*/or MSI analysis (as shown in **Figures 5E–F**). The mutation frequency of core gene mutations of major signaling pathways (PI3K-Akt signaling pathway, MicroRNAs pathway, DNA damage repair, and tumor suppressor genes) in G1 was significantly higher than that in G3 (**Figure 5G**).

**Table 4:** Clinicopathological and molecular characteristics of G1, G2, and G3 in this study.

Clinicopathologic characteristics	Number of patients (N=3937)	G1: *RNF43* codon 659-mutated, Non-class1 *BRAF*-mutated, and MSI-H, N (%) (N=34)	G2: Not G1 and G3, N (%) (N=3816)	G3: *RNF43* Non-codon 659-mutated, Class 1 *BRAF*-mutated, and Non-MSI-H, N (%) (N=87)	P value (G1 vs. G2)	P value (G1 vs. G3)	P value (G2 vs. G3)
Age					<0.0001	<0.0001	0.189
Young (years <50)	1300 (33.02%)	1 (2.94%)	1261 (33.05%)	38 (43.68%)			
Intermediate (<70 years ≥50)	1647 (41.83%)	11 (32.35%)	1605 (42.06%)	31 (35.63%)			
Elder (years≥70)	963 (24.46%)	22 (64.71%)	923 (24.19%)	18 (20.69%)			
NA	27 (0.69%)	0 (0.00%)	27 (0.71%)	0 (0.00%)			
Gender					0.1876	0.2404	0.0647
Female	1845 (46.86%)	21 (61.76%)	1782 (46.70%)	42 (48.28%)			
Male	2030 (51.56%)	13 (38.24%)	1976 (51.78%)	41 (47.13%)			
NA	62 (1.57%)	0 (0.00%)	58 (1.52%)	4 (4.60%)			
Primary tumor location					<0.0001	<0.0001	0.541
Right	916 (23.27%)	30 (88.24%)	867 (22.72%)	19 (21.84%)			
Left	2090 (53.09%)	1 (2.94%)	2046 (53.62%)	43 (49.43%)			
NA	931 (23.65%)	3 (8.82%)	903 (23.66%)	25 (28.74%)			
TNM stage					<0.0001	0.0087	0.0855
I	425 (10.80%)	4 (11.76%)	406 (10.64%)	15 (17.24%)			
II	743 (18.87%)	18 (52.94%)	704 (18.45%)	21 (24.14%)			
III	1224 (31.09%)	10 (29.41%)	1190 (31.18%)	24 (27.59%)			
IV	1375 (34.93%)	1 (2.94%)	1352 (35.43%)	22 (25.29%)			
NA	170 (4.32%)	1 (2.94%)	164 (4.30%)	5 (5.75%)			
TUMOR_GRADE					<0.0001	0.0767	0.152
Well differentiated	509 (12.93%)	4 (11.76%)	495 (12.97%)	10 (11.49%)			
Moderately differentiated	1351 (34.32%)	6 (17.65%)	1318 (34.54%)	27 (31.03%)			
Moderate poorly differentiated	110 (2.79%)	1 (2.94%)	105 (2.75%)	4 (4.60%)			
Poorly differentiated	333 (8.46%)	13 (38.24%)	307 (8.05%)	13 (14.94%)			
NA	1634 (41.50%)	10 (29.41%)	1591 (41.69%)	33 (37.93%)			
TMB					<0.0001	<0.0001	<0.0001
TMB-H	858 (21.79%)	34 (100.00%)	789 (20.68%)	35 (40.23%)			
TMB-L	2745 (69.72%)	0 (0.00%)	2700 (70.75%)	45 (51.72%)			
NA	334 (8.48%)	0 (0.00%)	327 (8.57%)	7 (8.05%)			
MSI					<0.0001	<0.0001	0.898
MSI-H	34 (0.86%)	34 (100.00%)	0 (0.00%)	0 (0.00%)			
MSS	2834 (71.98%)	0 (0.00%)	2768 (72.54%)	66 (75.86%)			
NA	874 (22.20%)	8 (23.53%)	845 (22.14%)	21 (24.14%)			
*BRAF* status					<0.0001	<0.0001	>0.9999
*BRAF* mut	329 (8.36%)	34 (100.00%)	289 (7.57%)	6 (6.90%)			
*BRAF* wild-type	3608 (91.64%)	0 (0.00%)	3527 (92.43%)	81 (93.10%)			
*BRAF* mutation types					0.0008	<0.0001	<0.0001
Class 1	224 (5.69%)	34 (100.00%)	190 (4.98%)	0 (0.00%)			
Class 2	9 (0.23%)	0 (0.00%)	9 (0.24%)	0 (0.00%)			
Class 3	40 (1.02%)	0 (0.00%)	40 (1.05%)	0 (0.00%)			
NA	56 (1.42%)	0 (0.00%)	50 (1.31%)	6 (6.90%)			

## 4 Discussion

Colorectal cancer (CRC) is highly heterogeneous and has significant prognostic differences (3, 4). Prognostic prediction based on molecular characteristics has been reported in some studies, but MSI-H is the only target that has been promoted to clinical treatment guidelines (21). In this study, we obtained data from 4,028 CRC patients for an in-depth analysis of *RNF43* as a potential target. This analysis revealed significant differences in PFS and no significant differences in OS between patients with *RNF43*-mutated and *RNF43* wild-type. DNA combined with RNA-based joint analysis in the coad_cptac_2019 cohort suggests that *RNF43*-mutated/expression < 0 shared a similar mutation spectrum with the *RNF43*-mutated, and *RNF43* wild-type/expression > 0 had a similar mutation spectrum to the *RNF43* wild-type, which represents a more optimal choice for precise prognostic stratification. *RNF43* codon 659-mutated can be used as a prognostic indicator for CRC in this study, and *RNF43* codon 659-mutated combined with Non-class1 *BRAF*-mutated and MSI-H has the best prognosis. *RNF43* Non-codon 659-mutated combined with Class 1 *BRAF*-mutated and Non-MSI-H had the worst prognosis. We also found that *RNF43* codon 659-mutated is highly correlated with MSI-H and TMB-H, which is consistent with previous studies (13), and indicates that *RNF43* codon 659-mutated is a special subtype and may be an advantageous subtype for ICIs. This study integrates all published CRC cohorts with clinicopathological and mutational information in Cbioport. To our knowledge, this is the largest CRC research dataset to date.

*RNF43* and *BRAF* are molecular events involved in the serrated tumor pathway during CRC development (31). Studies have reported that *RNF43* (24), *BRAF*, and MSI status have clear clinical significance at present. *RNF43*-mutated patients are associated with improved survival in CRC patients receiving ICIs (30). *RNF43*-mutated often co-occur with *BRAF* V600E mutations. The combination of *RNF43*-mutated with *BRAF* V600E mutations was significantly associated with poorer survival (20, 30). However, the above study did not provide a more detailed analysis of *RNF43*-mutated types or characteristics. At present, only one study divided *RNF43* into N-terminal and C-terminal based on codon 313 as a cutoff to demarcate the RING region, and found that *RNF43* mutations in the N-terminal region showed a shorter overall survival (19). *RNF43*, a WNT signaling pathway negative regulator, can predict the response of *BRAF* V600E MSS metastatic colorectal cancer against *BRAF*/*EGFR* combination therapy, where MSI-H always carries *RNF43* wildtype-like, encoding p.G659fs* and presents an intermediate response frequency (30). This suggests that the *RNF43* codon 659-mutated is a special subtype that warrants further study. MSI-H/dMMR are identified as key biomarkers guiding treatment strategies and disease management in CRC, suggesting that mCRC patients benefit from immunotherapy. Furthermore, *RNF43*-mutated were frequent (12.9%) in precancerous lesions of ulcerative colitis (UC) patients and detectable in 24.4% of colitis-associated cancer patients. *RNF43*-mutated caused invasive CRC by aggravating and perpetuating inflammation due to impaired epithelial barrier integrity and pathogen control, and *RNF43* inactivated mutation was even sufficient to cause spontaneous intestinal inflammation, resulting in subsequent invasive carcinoma development (32). Currently, there is no reference regarding the temporal sequence of *RNF43* and MSI-H. In 2018, a study established a 20-gene panel that could distinguish CRC from adenomas (33). In 2024, a study compared the mutation characteristics of different precancerous lesions and stage I-IV CRC. However, the role of *RNF43* in the process from precancerous lesions to the onset of CRC was not mentioned (34). Considering the high correlation between *RNF43* and MSI-H found in this study, it is of great significance to conduct DNA and RNA multi-omics exploration through gastroscopy polyp screening and hereditary tumor screening to deeply analyze the role of *RNF43* in the process from precancerous lesions to the onset of CRC. In this study, based on *RNF43* codon 659-mutated combined with Non-class1 *BRAF*-mutated or MSI-H, CRC has a better prognosis. *RNF43* codon 659-mutated combined with non-class 1 *BRAF*-mutated and MSI-H had the best prognosis, with OS reaching 100% in 13 patients, mainly stage III-IV CRC patients (11/13). It is also currently unreported that a combined biomarker can predict patient outcomes.

However, there are some limitations to this study. First of all, the data in this study came from a public database and only included SNV data, without collating CNV and SV data, which may provide obstacles for further findings, but the conclusions obtained in the current study will not be affected. Meanwhile, the completeness of the data we evaluated before the start of this study (**Supplementary Table 4**), the deletion rates further reinforces the reliability of the data and the conclusions drawn from it. Secondly, our study mainly provided cross-sectional data for the analysis of overall survival. Due to data limitations, we did not further analyze the subgroup of efficacy prediction based on the findings, which limited the innovation of this study. Subsequent studies should conduct efficacy prediction analysis in the cohort receiving targeted therapy and immunotherapy to verify the conclusions of this study and improve the depth of the overall study. Third, ethnic information was not available in this study, so the mutation heterogeneity among different ethnic groups was not deeply considered, which may limit the universality of the study’s conclusions. Fourth, the comparative analysis of *RNF43* wild-type and *RNF43*-mutated on the basis of MSI-H showed that OS had a trend of prognostic prediction. However, no statistically significant difference was found. Therefore, we cannot conclusively determine that *RNF43* is a key driver gene compared to MSI-H. Meanwhile, we only conducted independent cohort validations of the mutant and wild types of *RNF43*, and our findings were consistent with those from the analysis cohort. However, due to the limited number of validation codons, we have not yet performed cohort validations for the *RNF43* codon 659-mutated, which may affect the credibility of our results. In the future, under the condition of sufficient sample size and relatively fewer confounding factors, the mutation difference and prognosis difference of different populations can be compared to make up for the shortcomings of this study. In conclusion, subsequent studies can set up independent subgroups based on the findings of this study to improve the statistical robustness of the conclusions.

## 5 Conclusions

In conclusion, our findings elucidated a good prognosis of *RNF43* codon 659-mutated and concomitant Non-class 1 *BRAF*-mutated with MSI-H in CRC. Specifically, we found that *RNF43* codon 659-mutated is a specific subtype that is more likely to benefit from ICIs in CRC, causing the incidence of TMB-H and MSI-H in the *RNF43* codon 659-mutated group to be significantly higher. These results provided novel insights into the clinical applications based on mutation-based molecular typing that can help fine-screen populations with different prognoses and benefit from precision therapy.

## Data Availability

The original contributions presented in the study are included in the article/**Supplementary Material**. Further inquiries can be directed to the corresponding author.

## Supplementary material

Supplementary Figure 1 Lollipop plots (maps mutations on a linear protein and its domains) in each single cohort. Truncating includes frameshift mutations and nonsense mutations.

Supplementary Figure 2 A panoramic analysis of the genomic characteristics of *RNF43* wild-type in CRC. **(A)**: Top 50 mutation spectrum in *RNF43* wild-type patients. Each column represents a patient, and each row represents a gene. The table on the left represents the mutation rate of each gene. The top plot represents the overall number of mutations a patient carried. Different colors denote different types of mutations. **(B)**: KM analysis of PFS between *RNF43*-mutated and *RNF43* wild-type in this study. **(C)**: KM analysis of OS between *RNF43*-mutated and *RNF43* wild-type in this study. **(D)**: *RNF43* RNA expression levels in the *RNF43*-mutated and *RNF43* wild-type. **(E)**: The mutation landscape in the coad_cptac_2019 data set. CRC, Colorectal cancer; PFS: progression-free survival; OS, overall survival; KM, Kaplan-Meier.

Supplementary Figure 3 A panoramic analysis of the genomic characteristics of *RNF43* wild-type in validation cohort. A: Top 50 mutation spectrum in *RNF43*-mutated patients in validation cohort. B: Top 50 mutation spectrum in *RNF43* wild-type patients in validation cohort. Each column represents a patient, and each row represents a gene. The table on the left represents the mutation rate of each gene. The top plot represents the overall number of mutations a patient carried. Different colors denote different types of mutations. C: The differences in core gene mutation of major signaling pathways (PI3K-Akt signaling pathway, MicroRNAs pathway, DNA damage repair, and tumor suppressor genes) between *RNF43*-mutated and *RNF43* wild-type. D: KEGG functional enrichment analyses of *RNF43*-mutated and *RNF43* wild-type. E: GO functional enrichment analyses of *RNF43*-mutated and *RNF43* wild-type. GO, Gene Ontology; KEGG, Kyoto Encyclopedia of Genes and Genomes. *, p<0.05; **, p<0.01; ***, p<0.001.

Supplementary Figure 4 The mutation landscape analysis of the MSI-H subgroup with *RNF43*. A: The mutation landscape of the MSI-H and *RNF43*-mutated group. B: The mutation landscape of the MSI-H and *RNF43* wild-type group. C: The mutation landscape of the MSI-H and *RNF43* codon 659-mutated group. D: The mutation landscape of MSI-H and *RNF43* Non-codon 659-mutated group.

Supplementary Figure 5 A panoramic analysis of the survival outcome, genomic and pathway characteristics of *RNF43*-mutated, and *BRAF* in CRC. A: Top 50 mutation spectrum in *RNF43* codon 659-mutated and Non-class 1 *BRAF*-mutated patients. B: Top 50 mutation spectrum in *RNF43* Non-codon 659-mutated and Class 1 *BRAF*-mutated patients. Each column represents a patient, and each row represents a gene. The table on the left represents the mutation rate of each gene. The top plot represents the overall number of mutations a patient carried. Different colors denote different types of mutations. C: The volcanic maps for between *RNF43* codon 659-mutated/Non-class 1 *BRAF*-mutated patients and *RNF43* Non-codon 659-mutated/Class 1 *BRAF*-mutated. KEGG (D) and GO (E) functional enrichment analyses of *RNF43* codon 659-mutated/Non-class 1 *BRAF*-mutated patients and *RNF43* Non-codon 659-mutated/Class 1 *BRAF*-mutated patients. GO, Gene Ontology; KEGG, Kyoto Encyclopedia of Genes and Genomes. F: The differences in core gene mutation of major signaling pathways (PI3K-Akt signaling pathway, MicroRNAs pathway, DNA damage repair, and tumor suppressor genes) between *RNF43* codon 659-mutated/Non-class 1 *BRAF*-mutated patients and *RNF43* Non-codon 659-mutated/Class 1 *BRAF*-mutated.

Supplementary Figure 6 A panoramic analysis of the survival outcome, genomic and pathway characteristics of *RNF43*-mutated and *BRAF*-mutated in CRC. A: Top 50 mutation spectrum in *RNF43* codon 659-mutated and MSI-H patients. B: Top 50 mutation spectrum in *RNF43* Non-codon 659-mutated and Non-MSI-H patients. Each column represents a patient, and each row represents a gene. The table on the left represents the mutation rate of each gene. The top plot represents the overall number of mutations a patient carried. Different colors denote different types of mutations. MSI-H: patients with high MSI. C: The volcanic maps for between *RNF43* codon 659-mutated/MSI-H patients and *RNF43* Non-codon 659-mutated/Non-MSI-H patients. KEGG (D) and GO (E) functional enrichment analyses of *RNF43* codon 659-mutated/MSI-H patients and *RNF43* Non-codon 659-mutated/Non-MSI-H. GO, Gene Ontology; KEGG, Kyoto Encyclopedia of Genes and Genomes. F: The differences in core gene mutation of major signaling pathways (PI3K-Akt signaling pathway, MicroRNAs pathway, DNA damage repair, and tumor suppressor genes) between *RNF43* codon 659-mutated/MSI-H patients and *RNF43* Non-codon 659-mutated/Non-MSI-H.

## Glossary

CRC: Colorectal cancer
OS: Overall Survival
PFS: Progression-free survival
ICIs: Immune checkpoint inhibitors
NCCN: the National Comprehensive Cancer Network
RNF43: Ring finger protein 43
BRAF: B-Raf proto-oncogene, serine/threonine kinase
MAPK: the Mitogen-Activated Protein Kinase
MSI: Microsatellite instability
MSS: Microsatellite stable
TMB: Tumor mutational burden
TNM: The Tumor-Node-Metastasis system
HR: Hazard ratio
CI: Confidence interval
PI3K: Phosphoinositol-3 kinase
TCGA: The Cancer Genome Atlas
cBioPortal: cBio Cancer Genomics Portal
GO: Gene Ontology
KEGG: Kyoto Encyclopedia of Genes and Genomes
KM: Kaplan-Meier
ARID1A: AT-rich interaction domain 1A
CIC: Capicua transcriptional repressor
PIK3CA: Phosphatidylinositol-4,5-bisphosphate 3-kinase catalytic subunit alpha
PTPRS: Protein tyrosine phosphatase receptor type S
APC: APC regulator of WNT signaling pathway
FAT1: FAT atypical cadherin 1
POLE: DNA polymerase epsilon, catalytic subunit
NOTCH3: Notch receptor 3
SPEN: Spen family transcriptional repressor
TP53: Tumor protein p53
KRAS: KRAS proto-oncogene, GTPase
FBXW7: F-box and WD repeat domain containing 7
SMAD4: SMAD family member 4
TCF7L2: Transcription factor 7 like 2
SOX9: SRY-box transcription factor 9
PTCH1: Patched 1
SMARCA4: SWI/SNF related BAF chromatin remodeling complex subunit ATPase 4
FLT4: Fms related receptor tyrosine kinase 4
