# Supplementary figures and images for: Define a good prognosis of RNF43 codon 659-mutated and concomitant genomic signatures in CRC: an analysis of the cBioPortal database
Source: Front Oncol. 2025 Aug 8;15:1608664. doi: 10.3389/fonc.2025.1608664 (PMC12371537; doi:10.3389/fonc.2025.1608664)

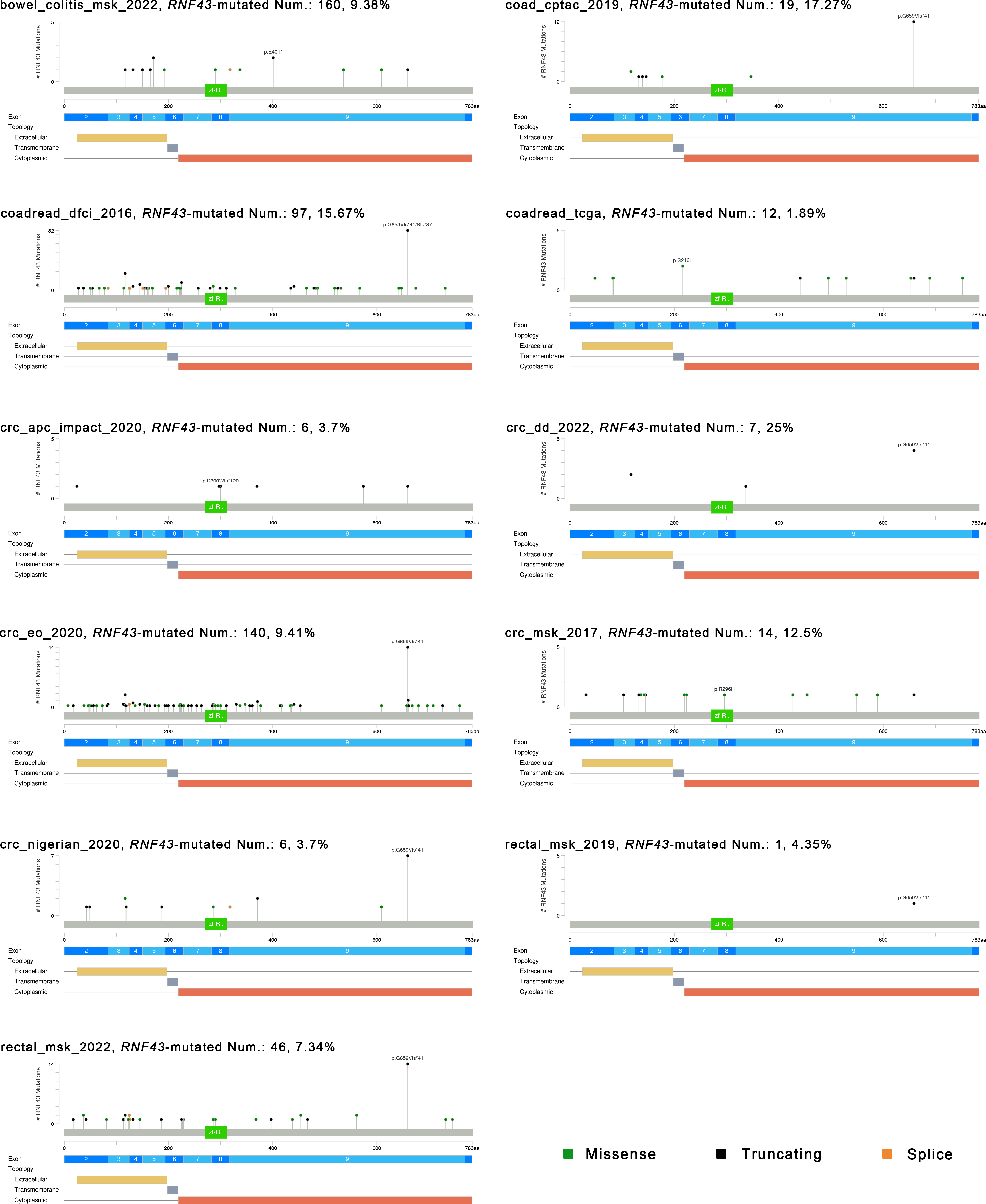

Supplement: Supplementary Figure 1 — Lollipop plots (maps mutations on a linear protein and its domains) in each single cohort. Truncating includes frameshift mutations and nonsense mutations. [file Image1.tif]

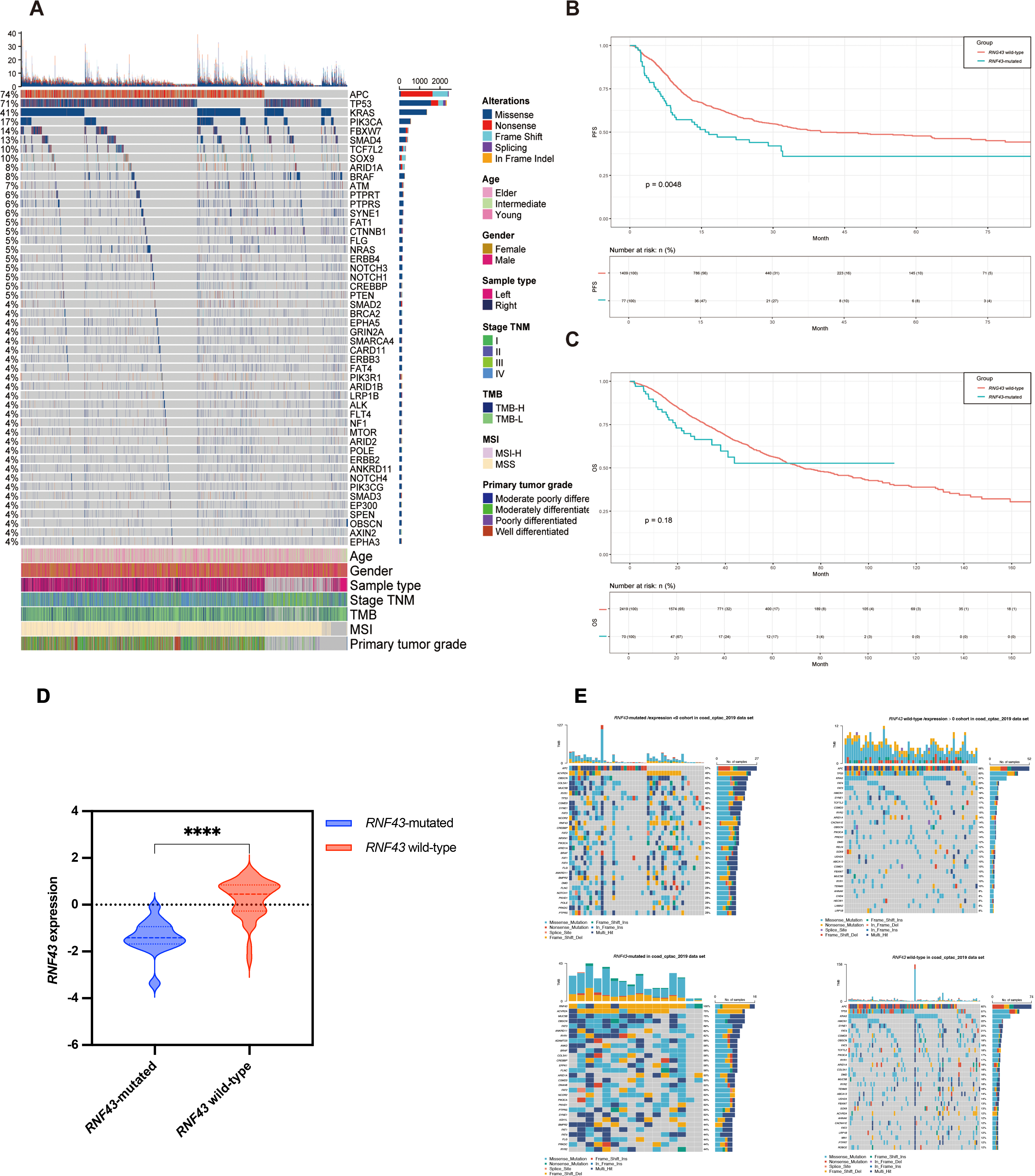

Supplement: Supplementary Figure 2 — A panoramic analysis of the genomic characteristics of RNF43 wild-type in CRC. (A): Top 50 mutation spectrum in RNF43 wild-type patients. Each column represents a patient, and each row represents a gene. The table on the left represents the mutation rate of each gene. The top plot represents the overall number of mutations a patient carried. Different colors denote different types of mutations. (B): KM analysis of PFS between RNF43-mutated and RNF43 wild-type in this study. (C): KM analysis of OS between RNF43-mutated and RNF43 wild-type in this study. (D): RNF43 RNA expression levels in the RNF43-mutated and RNF43 wild-type. (E): The mutation landscape in the coad_cptac_2019 data set. CRC, Colorectal cancer; PFS: progression-free survival; OS, overall survival; KM, Kaplan-Meier. [file Image2.tif]

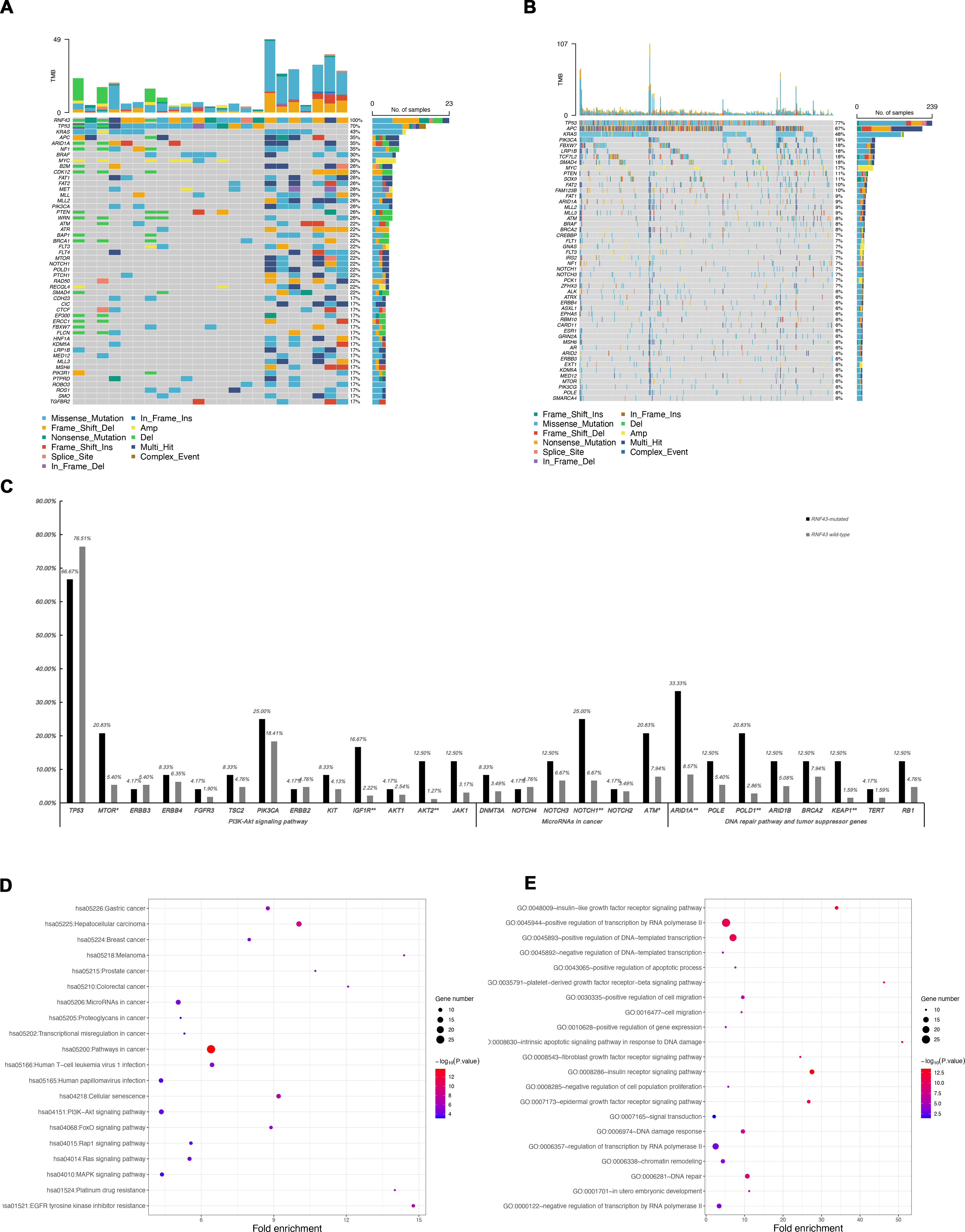

Supplement: Supplementary Figure 3 — A panoramic analysis of the genomic characteristics of RNF43 wild-type in validation cohort. A: Top 50 mutation spectrum in RNF43-mutated patients in validation cohort. B: Top 50 mutation spectrum in RNF43 wild-type patients in validation cohort. Each column represents a patient, and each row represents a gene. The table on the left represents the mutation rate of each gene. The top plot represents the overall number of mutations a patient carried. Different colors denote different types of mutations. C: The differences in core gene mutation of major signaling pathways (PI3K-Akt signaling pathway, MicroRNAs pathway, DNA damage repair, and tumor suppressor genes) between RNF43-mutated and RNF43 wild-type. D: KEGG functional enrichment analyses of RNF43-mutated and RNF43 wild-type. E: GO functional enrichment analyses of RNF43-mutated and RNF43 wild-type. GO, Gene Ontology; KEGG, Kyoto Encyclopedia of Genes and Genomes. *, p<0.05; **, p<0.01; ***, p<0.001. [file Image3.tif]

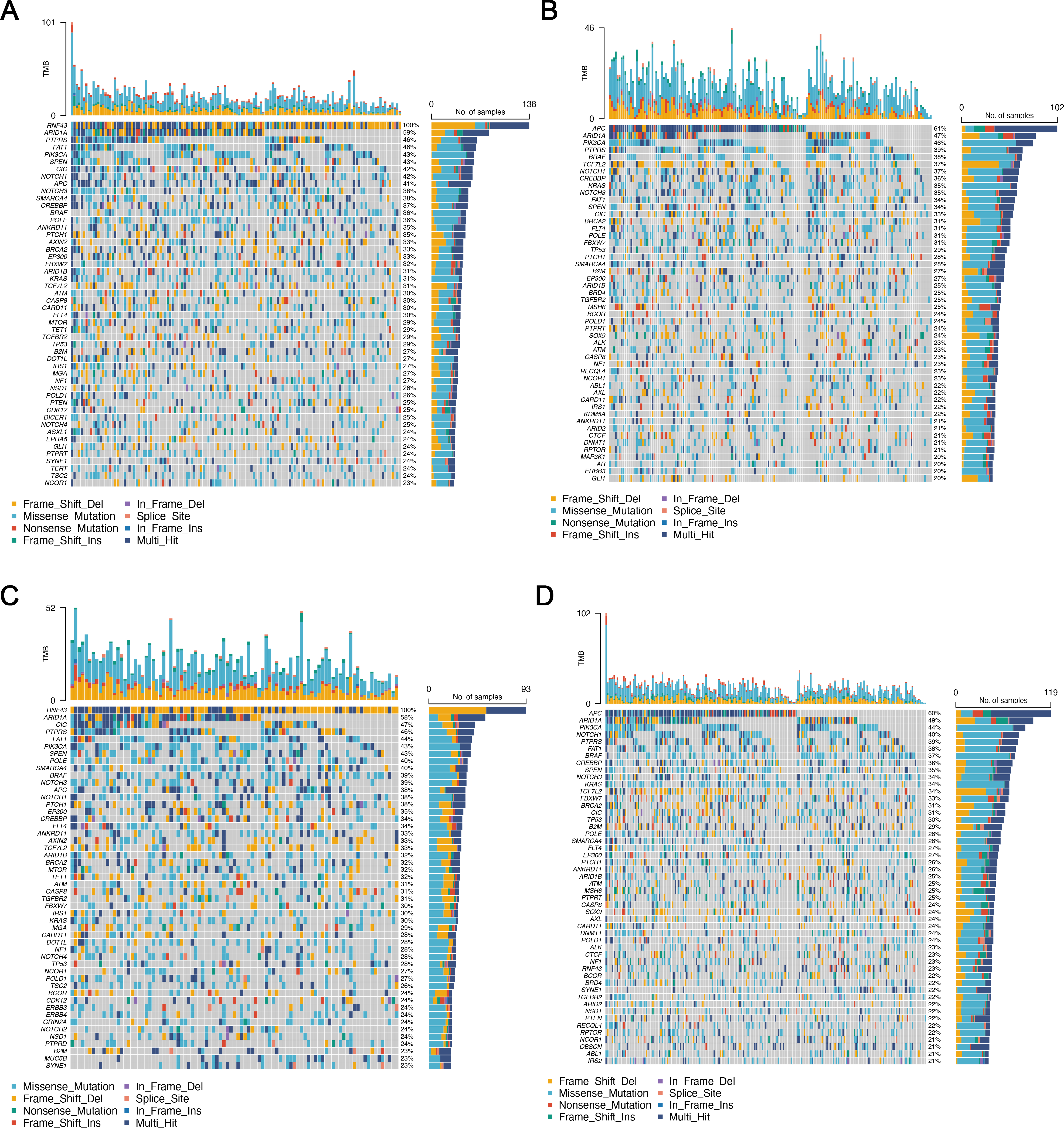

Supplement: Supplementary Figure 4 — The mutation landscape analysis of the MSI-H subgroup with RNF43. A: The mutation landscape of the MSI-H and RNF43-mutated group. B: The mutation landscape of the MSI-H and RNF43 wild-type group. C: The mutation landscape of the MSI-H and RNF43 codon 659-mutated group. D: The mutation landscape of MSI-H and RNF43 Non-codon 659-mutated group. [file Image4.tif]

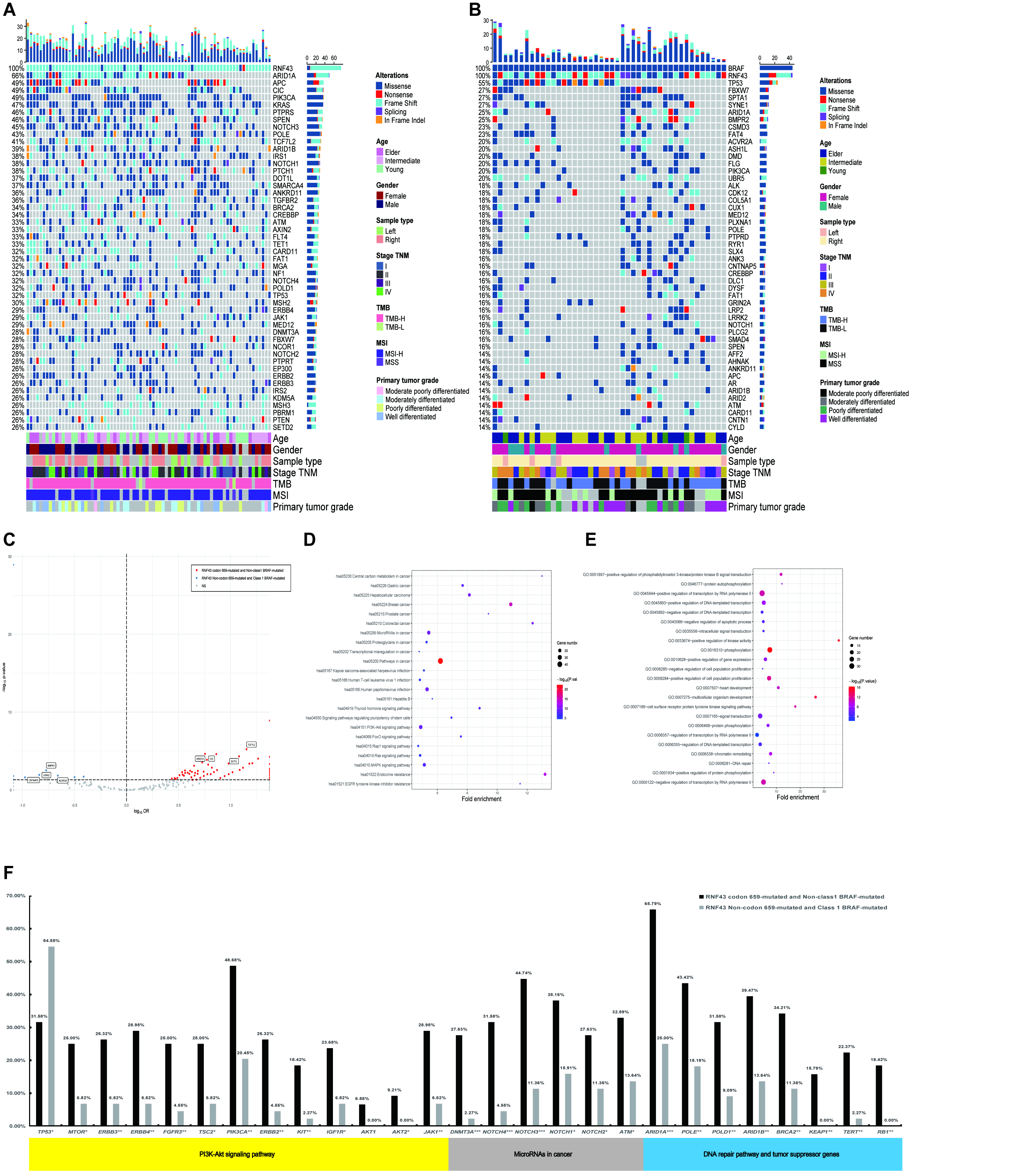

Supplement: Supplementary Figure 5 — A panoramic analysis of the survival outcome, genomic and pathway characteristics of RNF43-mutated, and BRAF in CRC. A: Top 50 mutation spectrum in RNF43 codon 659-mutated and Non-class 1 BRAF-mutated patients. B: Top 50 mutation spectrum in RNF43 Non-codon 659-mutated and Class 1 BRAF-mutated patients. Each column represents a patient, and each row represents a gene. The table on the left represents the mutation rate of each gene. The top plot represents the overall number of mutations a patient carried. Different colors denote different types of mutations. C: The volcanic maps for between RNF43 codon 659-mutated/Non-class 1 BRAF-mutated patients and RNF43 Non-codon 659-mutated/Class 1 BRAF-mutated. KEGG (D) and GO (E) functional enrichment analyses of RNF43 codon 659-mutated/Non-class 1 BRAF-mutated patients and RNF43 Non-codon 659-mutated/Class 1 BRAF-mutated patients. GO, Gene Ontology; KEGG, Kyoto Encyclopedia of Genes and Genomes. F: The differences in core gene mutation of major signaling pathways (PI3K-Akt signaling pathway, MicroRNAs pathway, DNA damage repair, and tumor suppressor genes) between RNF43 codon 659-mutated/Non-class 1 BRAF-mutated patients and RNF43 Non-codon 659-mutated/Class 1 BRAF-mutated. [file Image5.tif]

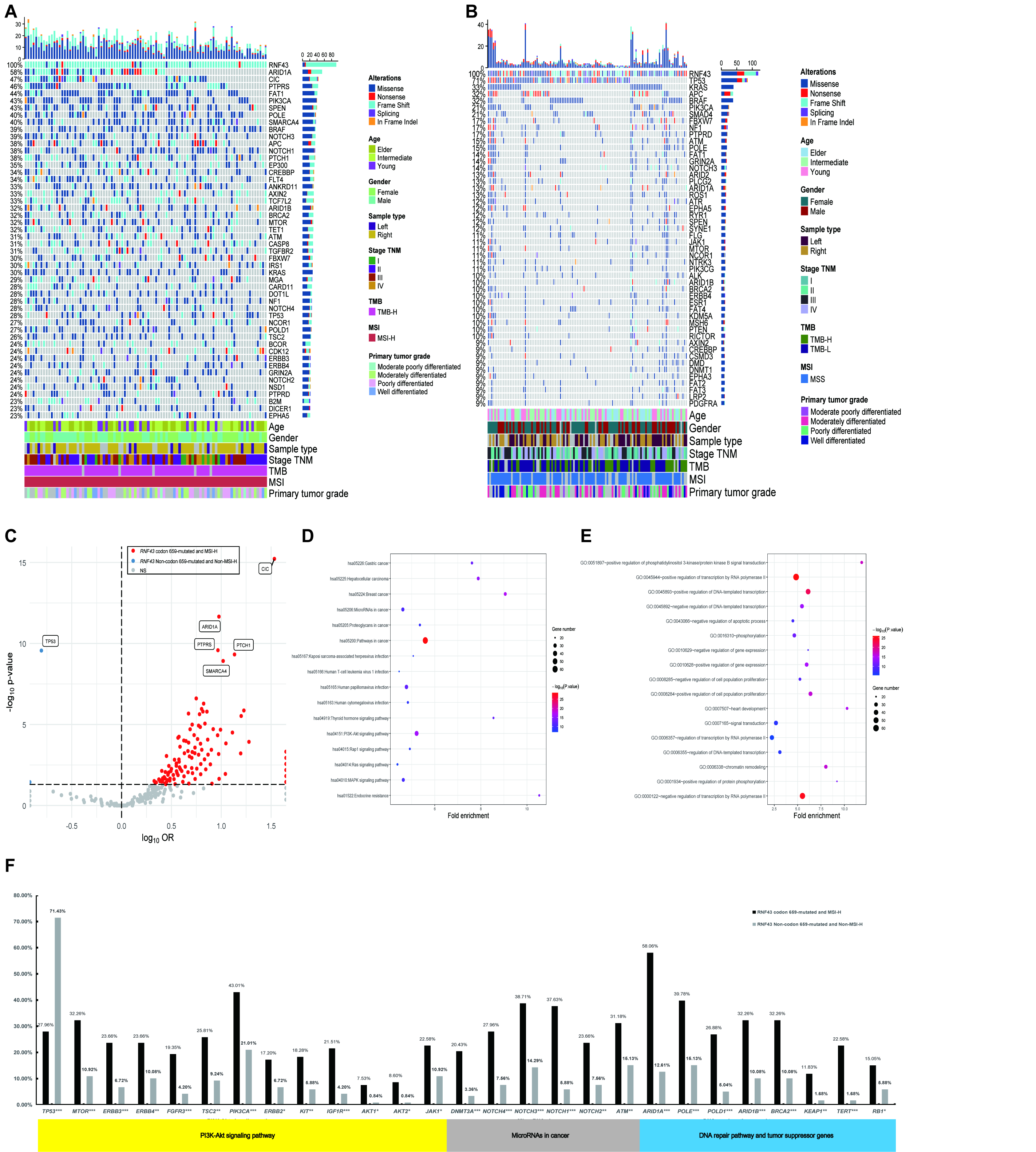

Supplement: Supplementary Figure 6 — A panoramic analysis of the survival outcome, genomic and pathway characteristics of RNF43-mutated and BRAF-mutated in CRC. A: Top 50 mutation spectrum in RNF43 codon 659-mutated and MSI-H patients. B: Top 50 mutation spectrum in RNF43 Non-codon 659-mutated and Non-MSI-H patients. Each column represents a patient, and each row represents a gene. The table on the left represents the mutation rate of each gene. The top plot represents the overall number of mutations a patient carried. Different colors denote different types of mutations. MSI-H: patients with high MSI. C: The volcanic maps for between RNF43 codon 659-mutated/MSI-H patients and RNF43 Non-codon 659-mutated/Non-MSI-H patients. KEGG (D) and GO (E) functional enrichment analyses of RNF43 codon 659-mutated/MSI-H patients and RNF43 Non-codon 659-mutated/Non-MSI-H. GO, Gene Ontology; KEGG, Kyoto Encyclopedia of Genes and Genomes. F: The differences in core gene mutation of major signaling pathways (PI3K-Akt signaling pathway, MicroRNAs pathway, DNA damage repair, and tumor suppressor genes) between RNF43 codon 659-mutated/MSI-H patients and RNF43 Non-codon 659-mutated/Non-MSI-H. [file Image6.tif]
